# Fbxw11 promotes the proliferation of lymphocytic leukemia cells through the concomitant activation of NF-κB and β-catenin/TCF signaling pathways

**DOI:** 10.1038/s41419-018-0440-1

**Published:** 2018-03-19

**Authors:** Lina Wang, Wenli Feng, Xiao Yang, Feifei Yang, Rong Wang, Qian Ren, Xiaofan Zhu, Guoguang Zheng

**Affiliations:** State Key Laboratory of Experimental Hematology, Institute of Hematology and Blood Diseases Hospital, Chinese Academy of Medical Sciences & Peking Union Medical College, 288 Nanjing Road, 300020 Tianjin, China

## Abstract

The ubiquitin–proteasome system (UPS) participates in both physiological and pathological processes through the posttranslational regulation of intracellular signal transduction pathways. F-box and WD-40 domain protein 11 (Fbxw11) is a component of the SCF (Skp1–Cul1–F-box) E3 ubiquitin ligase complex. Fbxw11 regulates various signal transduction pathways, and it may have pathological roles in tumorigenesis. However, the role of Fbxw11 in the development of leukemia and the underlying mechanisms remain largely unknown. In this study, Fbxw11 expression was aberrantly upregulated in patients with lymphocytic leukemia. Its expression was dramatically decreased in patients who achieved complete remission (CR) after chemotherapy. The high level of Fbxw11 expression in L1210 lymphocytic leukemia cells stimulated cell proliferation in vitro and tumor formation in vivo. The effects were mediated by the stimulation of cell cycle progression rather than the induction of apoptosis. Furthermore, a bioinformatics analysis suggested concomitant activation of the NF-κB and β-catenin/TCF signaling pathways, which were confirmed by reporter gene assays. Moreover, blocking experiments suggested the involvement of both pathways in the growth-promoting effects of Fbxw11. Our results reveal the role of Fbxw11 in lymphocytic leukemia cells and imply that Fbxw11 may serve as a potential molecular target for the treatment of lymphocytic leukemia.

## Introduction

Hematopoiesis is strictly regulated by complicated intercellular communication from the hematopoietic microenvironment through sophisticated signal transduction networks. Dysregulation of signal transduction will disrupt the balance of normal hematopoiesis and cause various blood diseases. Leukemia is regarded as a clonal disease^1,2^, and many intrinsic and extrinsic factors have been verified to play parts in the initiation and development of leukemia^3,4^. During leukemogenesis, leukemia cells outcompete their normal counterparts and become dominant due to their high capacity for self-renewal and low capacity for apoptosis^5^. Diverse intrinsic abnormalities, which endow leukemia cells with those characteristics, have been elucidated at different levels, including mRNA transcriptional control, protein translation, and posttranslational modifications^6–10^.

The ubiquitin–proteasome system (UPS), which is the main pathway for the degradation of short-period proteins in cells, is involved in the posttranslational regulation of numerous intracellular signal transduction pathways^11^. The Skp1/cullin/F-box (SCF) complex is an important E3 ubiquitin ligase. F-box family proteins, which are further divided into Fbxw, Fbxl, and Fbxo subfamilies based on protein structure, determine the specificity of substrate degradation by identifying and binding to different target proteins^12^. Abnormal expression or dysfunction of several F-box proteins results in aberrant ubiquitination, inducing the development and progression of malignancies, including hematopoietic malignancies. Some F-box family members contribute to tumorigenesis and tumor development^13–15^. Fbxw7 controls leukemia-initiating cells in chronic myelogenous leukemia and chronic myeloid leukemia (CML) by regulating c-Myc ubiquitination^16–18^. Fbxo11 loss or mutation induces impairments in BCL6 degradation, and therefore BCL6 accumulation contributes to pathogenesis of diffuse large B-cell lymphomas^19^. In addition, the E3 ligase family members Fbxl2, Fbxl10, and SKP2 participate in the proliferation of leukemia cells by regulating the ubiquitination pathway^20–22^. To date, the effects of other members in the F-box family on the development of hematopoietic malignancies have not been established.

F-box and WD repeat domain containing 11 (Fbxw11), also known as HOS or β-TrCP2, belongs to the Fbxw subfamily of the F-box protein family^23^. Fbxw11 is crucial for embryonic development, and the most obvious defect in Fbxw11^−/−^ mice is embryonic mortality^24^. Fbxw11 plays pivotal roles in various signaling pathways by regulating the ubiquitination of phosphorylated substrates. The SCF^Fbxw11^ complex regulates many important biological processes, including the cell cycle, differentiation, development, and metabolism, by targeting a broad range of substrates, including IκB, β-catenin, ATF4, Emi1, etc.^25–29^. Fbxw11 recognizes and binds to phosphorylated IκB and β-catenin, which triggers their degradation through the UPS. The nuclear factor (NF)-κB and Wnt/β-catenin signaling pathways are closely associated with hematopoiesis^30,31^. Studies of the mechanisms by which Fbxw11 regulates the development and progression of solid tumors have mainly focused on the activation of the NF-κB pathway. Fbxw11 plays an important role in controlling the IκB-dependent apoptotic pathway in human melanoma^32^. Furthermore, Fbxw11 is overexpressed in mouse skin tumors and accelerates tumor progression by activating the NF-κB signaling pathway^33^. Moreover, associations among Fbxw11, β-catenin, and NF-κB have been observed in colorectal cancer^32^. In our previous study, upregulation of Fbxw11 in hematopoietic stem progenitor cells (HSPCs) in the T-cell acute lymphocytic leukemia (T-ALL) microenvironment was detected. According to the results of a preliminary study, Fbxw11 expression was upregulated in bone marrow (BM) samples from patients with acute lymphocytic leukemia (ALL). However, the role of Fbxw11 in leukemia development remains largely unknown.

In the present study, we examined the expression of Fbxw11 in leukemia samples and explored the role of Fbxw11 in lymphocytic leukemia cells using in vitro and in vivo experiments. Fbxw11 expression was upregulated in ALL samples. Furthermore, the high-level expression of three variants of the Fbxw11 transcript in the lymphocytic leukemia cell line L1210 accelerated proliferation in vitro and promoted tumor formation in vivo. Moreover, Fbxw11-mediated concomitant activation of the NF-κB and β-catenin/T-cell factor (TCF) signaling pathways contributed to increased proliferation in lymphocytic leukemia cells.

## Results

### Fbxw11 expression in hematopoietic malignancies

**Fig. 1:**
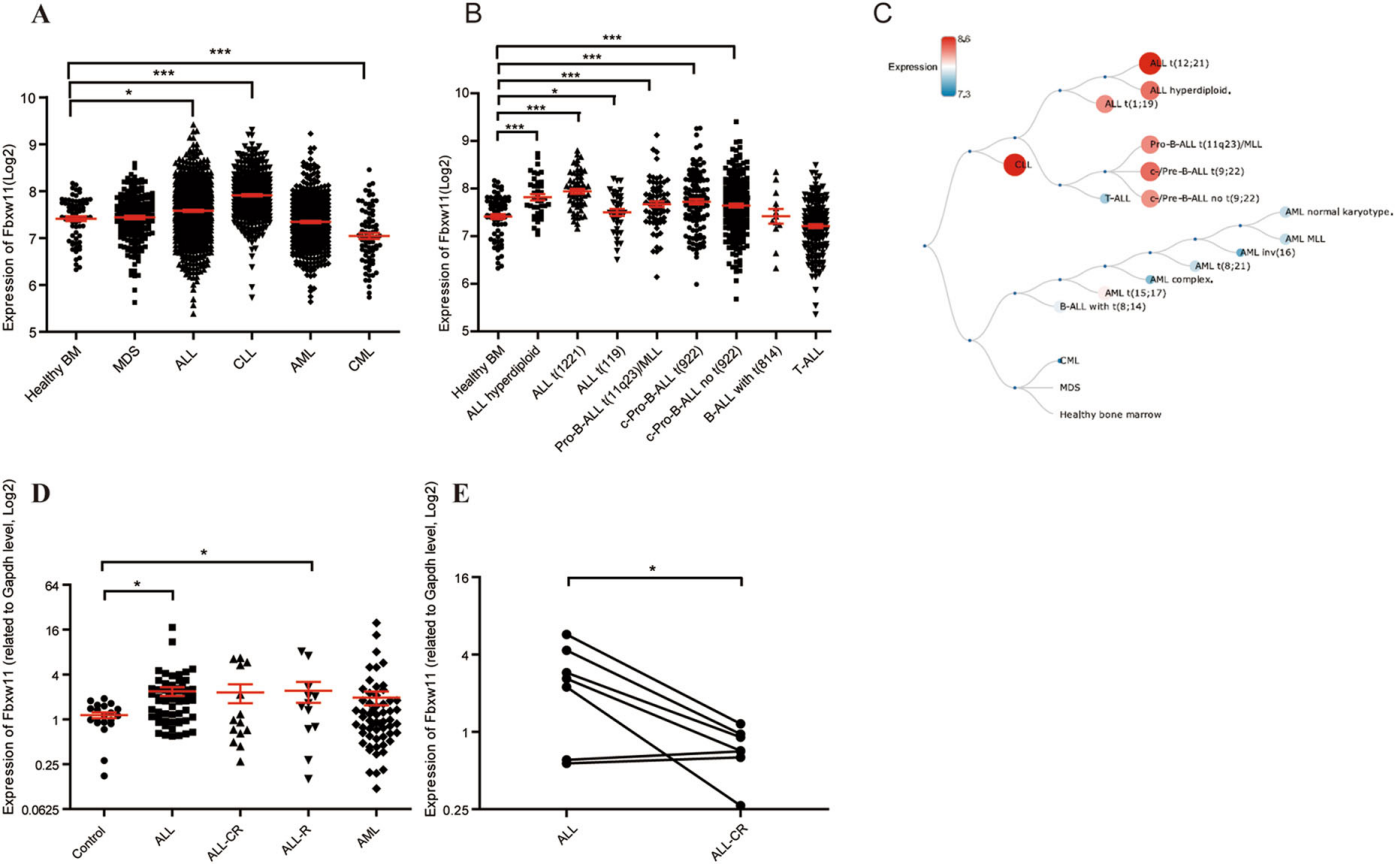
Fbxw11 expression in hematopoietic malignancies. **a**–**c** Fbxw11 expression was analyzed in the GSE13159 dataset (*n* = 2095) from MILE in the BloodSpot database. Fbxw11 expression among AML, ALL, CML, CLL, MDS, and healthy BM samples (**a**) and among ALL cytogenetic subtypes (**b**) are shown. The Tree diagram shows differences in the Fbxw11 expression among patients with different types of leukemia (**c**). **d**, **e** Fbxw11 expression in BMMCs was detected using real-time PCR. **d** Fbxw11 expression in 20 control samples and 146 pediatric patients with AL, including newly diagnosed ALL (*n* = 60), ALL in complete remission (ALL-CR, *n* = 15), relapsed ALL (ALL-R, *n* = 12), and newly diagnosed AML (*n* = 59), is shown. **e** A follow-up study of Fbxw11 expression in 7 ALL patients before and after standard chemotherapy achieving CR. The real-time PCR results were obtained from three independent experiments. *p < 0.05, ***p < 0.001

Fbxw11 expression was initially analyzed in hematopoietic malignancies in the BloodSpot database to assess the clinical relevance of Fbxw11^34^. Based on the analysis of the GSE13159 dataset from Microarray Innovations in Leukemia (MILE), Fbxw11 was expressed at significantly higher levels in the ALL or chronic lymphocytic leukemia (CLL) group than the healthy BM group (Fig. 1a). The amplitude of the expression change was converted from log2 expression data. Compared with the healthy BM group, the average expression of Fbxw11 showed 12.23% increase in ALL group, 41.13% increase in CLL group and 22.57% decrease in CML group. Furthermore, in patients with ALL presenting with cytogenetic abnormalities, Fbxw11 was expressed at high levels observed in most subtypes, except for B-cell acute lymphocytic leukemia (B-ALL) with t(8;14) or T-ALL (Fig. 1b). Interactive hierarchical tree showed the difference of Fbxw11 expression among different types of leukemia (Fig. 1c).

Fbxw11 expression in patients with acute leukemia (AL) was investigated using real-time PCR. Fbxw11 was expressed at higher levels in patients with newly diagnosed ALL and relapsed ALL than in healthy donors (Fig. 1d). Fbxw11 was expressed at lower levels in patients with ALL who achieved complete remission (CR), although the difference was not statistically significant due to the limited number of cases and heterogeneity among patients (Fig. 1d). Then a follow-up study was performed in 7 cases of ALL patients to compare Fbxw11 expression in the same patients before and after standard chemotherapy achieving CR. The result revealed that Fbxw11 expression was decreased significantly in patients who achieved CR after chemotherapy (Fig. 1e). A statistical analysis was also performed on clinical features, including risk, age, TEL/AML1, chromosome translocation, BCR/ABL chromosome translocation, lactate dehydrogenase level, leukocyte count, and 7-day peripheral blood response to prednisone treatment. Statistically significant differences were not observed (Figure S1).

### High levels of Fbxw11 expression promoted the growth of L1210 cells in vitro

**Fig. 2:**
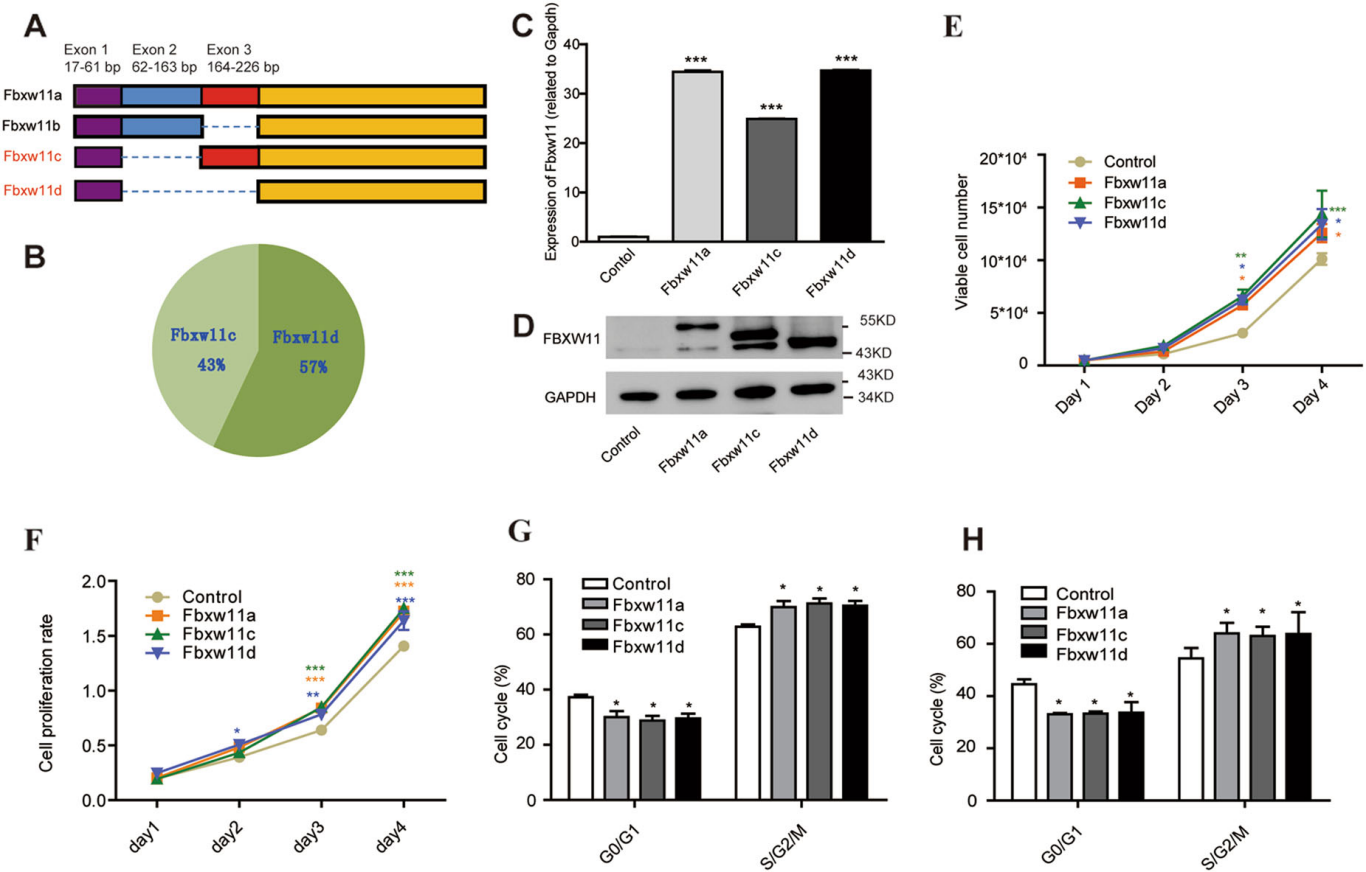
Fbxw11 promotes cell proliferation in vitro. **a** Schematic of the Fbxw11 gene. **b** Pie chart shows the proportion of transcript variants (Fbxw11c and Fbxw11d) expressed in mouse BM cells. **c**,** d** L1210 cell lines expressing high levels of Fbxw11 variants were established by retroviral infection and verified by real-time RT-PCR (**c**) and western blotting (**d**).** e**,** f** The proliferation potential of L1210 cells with or without high levels of Fbxw11 isoforms in vitro was assessed by counting cells (**e**) or the MTS method (**f**). **g** Changes in the cell cycle after Fbxw11 overexpression were detected by PI staining followed by FACS analysis. **h** Cell cycle analysis using Ki67 and Hoechst 33342 staining. Data are presented as means ± SEM (*n* = 3; 3 independent experiments). *p < 0.05, **p < 0.01, ***p < 0.001

Fbxw11 is expressed as three transcript variants in humans and four variants in mice through alternative splicing^24,35^. A schematic of mouse Fbxw11 is shown in Fig. 2a. Fbxw11a is the full-length transcript encoding the largest isoform. Fbxw11b and Fbxw11c contain an in-frame deletion of exon 3 or exon 2, respectively, whereas Fbxw11d lacks both exons. More than 30 transcripts were cloned from mouse BM samples covering only Fbxw11c (43%) and Fbxw11d (57%), suggesting that those two isoforms might be important for hematopoiesis (Fig. 2b). The L1210 lymphocytic leukemia cell line was chosen for further study and infected with retroviruses carrying Fbxw11 variants. Stably transfected cell lines were obtained by cell sorting and defined as L1210-control, L1210-Fbxw11a, L1210-Fbxw11c, and L1210-Fbxw11d, respectively. Cells expressing high levels of Fbxw11 variants were verified by real-time reverse transcriptase PCR (RT-PCR) and western blotting (Fig. 2c, d). Cell proliferation, apoptosis, and migration were investigated to assess the effects of Fbxw11 on leukemia cells in vitro. Fbxw11 had little effect on apoptosis (Figure S2). In contrast, high levels of Fbxw11 expression promoted the proliferation of L1210 cells. Based on the results of the growth curves obtained by counting cells (Fig. 2e) and the MTS (3-(4,5-dimethylthiazol-2-yl)-5-(3-carboxymethoxyphenyl)-2-(4-sulfophenyl)-2H-tetrazolium) assay (Fig. 2f), Fbxw11 significantly enhanced the growth of L1210 cells. A cell cycle analysis was performed using PI staining and Ki-67/Hoechst33342 staining. Typical fluorescence-activated cell sorrting results are shown (Figure S3). Increased percentages of L1210-Fbxw11a, L1210-Fbxw11c, and L1210-Fbxw11d cells in S/G2/M phase were detected using propidium iodide (PI) staining (Fig. 2g) and Ki-67/Hoechst33342 staining (Fig. 2h).

### High levels of Fbxw11 expression promoted the formation of tumors comprising L1210 cells in vivo

**Fig. 3:**
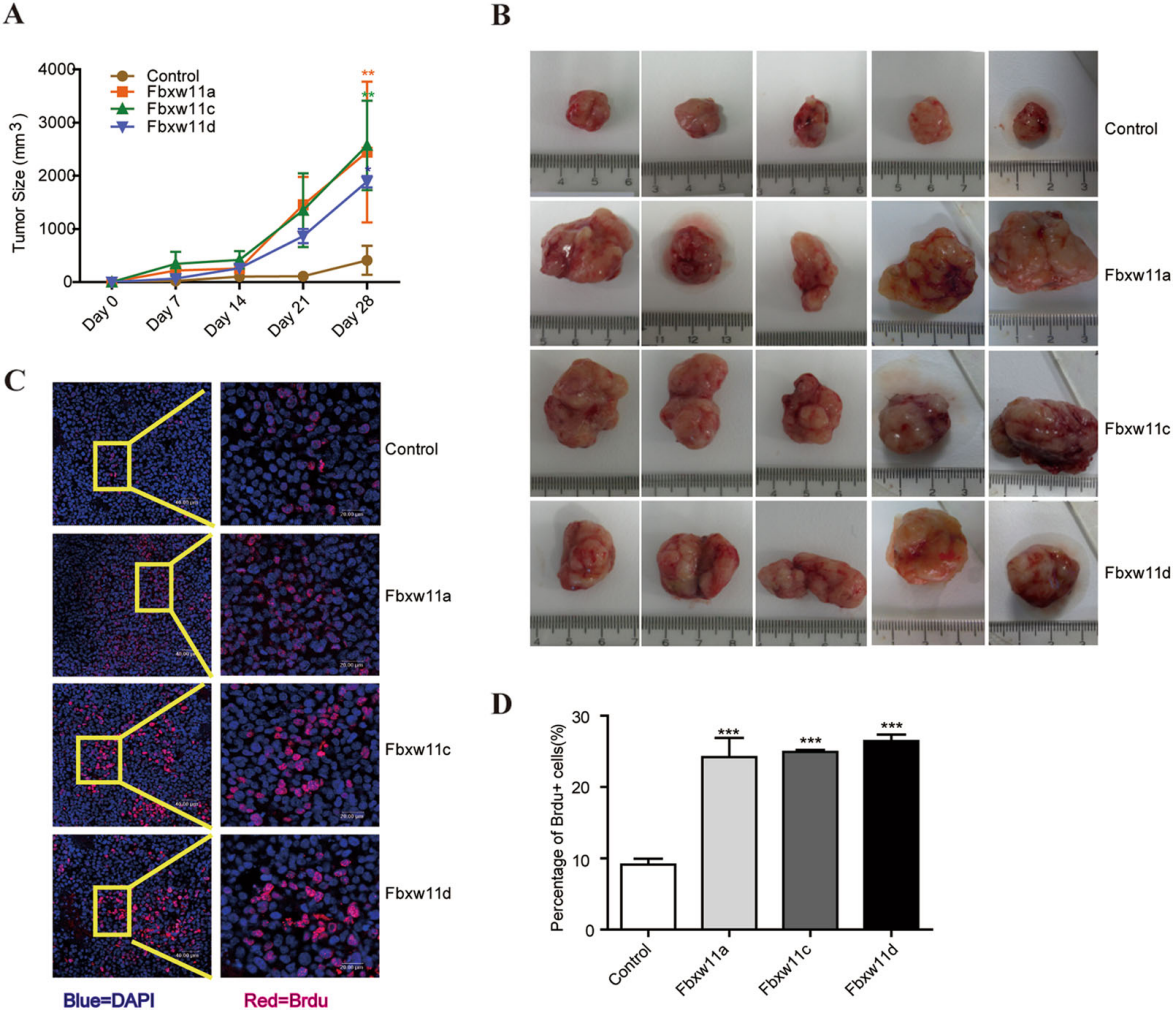
Fbxw11 promotes tumor formation in vivo. The dorsal surfaces of DBA2 mice were s.c. injected with leukemia cells. **a** Tumors were measured weekly after the leukemia cell injection. Tumor volumes were calculated using the formula length × width^2^/2. (*n* = 5). **b** Representative images of the tumors from mice sacrificed on day 28. Tumors expressing high levels of Fbxw11 were larger than control tumors. **c** The proliferation of L1210 cells with or without high levels of Fbxw11 expression was assessed using the BrdU incorporation assay. BrdU-positive cells were counted in at least five random fields using a microscope. Representative images of BrdU staining are shown. Scale bars represent 40 μm for low-magnification images and 20 μm for high-magnification images. Blue represents DAPI and red indicates BrdU. **d** Statistical analysis of the BrdU incorporation assay. Data are presented as means ± SEM (*n* = 5; 3 independent experiments). *p < 0.05, **p < 0.01, ***p < 0.001

DBA/2J mice were subcutaneously injected with 1 × 10^6^ L1210-control, L1210-Fbxw11a, L1210-Fbxw11c, and L1210-Fbxw11d cells (*n* = 5/group) to further examine the effects of Fbxw11 on the proliferation of leukemia cells in vivo. Tumor volumes were measured weekly and calculated using the formula: length × width^2^/2. Tumor growth was significantly increased in groups expressing high levels of Fbxw11 variants (Fig. 3a). Mice were sacrificed on day 28 and the tumors displaying high levels of Fbxw11 expression were larger than control tumors (Fig. 3b). Bromodeoxyuridine (BrdU) incorporation experiment showed a significantly higher rate of BrdU-positive cells in tumors formed by L1210 cells expressing high levels of Fbxw11 variants than control slides using confocal microscopy (Fig. 3c, d). Taken together, high levels of Fbxw11 transcript variants promoted L1210 cell growth in vitro and in vivo.

### Effects of Fbxw11 on gene expression profiles in L1210 cells

**Fig. 4:**
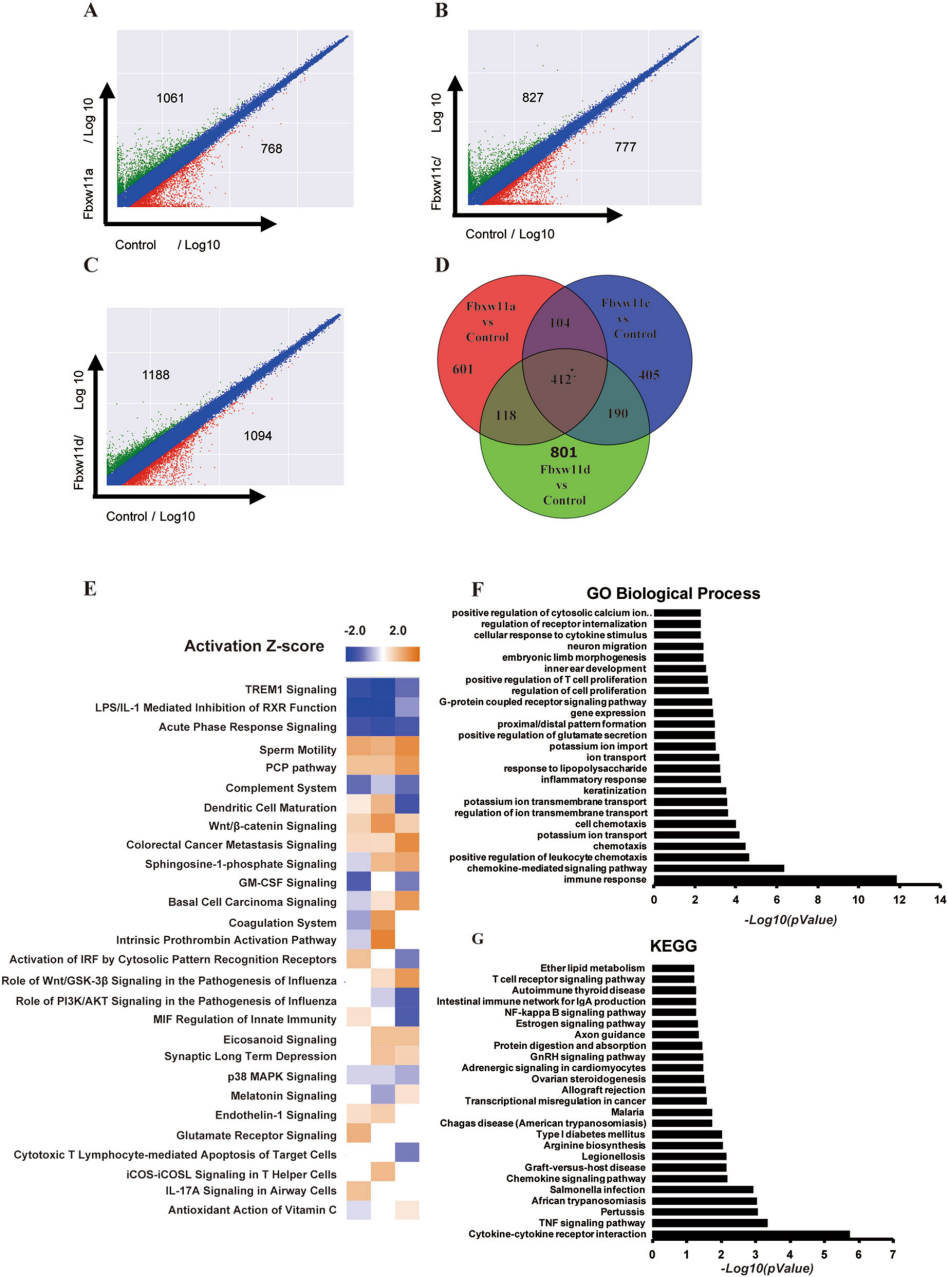
Fbxw11 affects gene expression profiles in L1210 cells. Total RNA from L1210-control, L1210-Fbxw11a, L1210-Fbxw11c, and L1210-Fbxw11d cells was applied to an Agilent Sureprint G3 Mouse Gene Expression Microarray analysis**. a**–**c** Scatter plots show the fold changes in gene expression in L1210 cells expressing high levels of Fbxw11 compared with L1210-control cells. **d** DEGs in L1210 cells expressing high levels of Fbxw11 were normalized to L1210-control cells and summarized; the Venn diagram illustrates the number of overlapping genes. **e** Canonical pathway analysis was performed based on DEGs from the normalized data of the three groups, which were standardized to control cells by using the IPA software. The IPA activation *z*-score algorithm was used to predict the direction of regulation for a biological process. A *z*-score ≥ 2.0 predicts a significant increase of a function or process, whereas a *z*-score ≤ -2.0 predicts a significant decrease. GO analysis (**f**) and KEGG pathway annotation analysis (**g**) of DEGs were performed using the FunNet database

Total RNA was isolated from L1210-control, L1210-Fbxw11a, L1210-Fbxw11c and L1210-Fbxw11d cells and applied to an Agilent Sureprint G3 Mouse Gene Expression Microarray to investigate the mechanism by which Fbxw11 transcript variants promoted proliferation. A fold change ≥2.0 was used as a cutoff. Based on the scatter plots, >1500 genes were differentially expressed in each L1210 cell line expressing high levels of an Fbxw11 transcript variant compared with L1210-control cells (Fig. 4a–c). Genes with fold changes >2 were selected for a Venn analysis to show differences and similarities among L1210-Fbxw11a, L1210-Fbxw11c, and L1210-Fbxw11d cells (Fig. 4d). Differentially expressed genes (DEGs) normalized to L1210-control cells were then analyzed using Ingenuity Pathways Analysis (IPA), which enables comparisons among different groups. The IPA activation *z*-score algorithm was used to predict the direction of regulation (increase if a *z*-score ≥ 2.0, decrease if a *z*-score ≤ -2.0) for a biological process. To determine the top canonical pathways associated with the observed differentially expressed gene, we performed a canonical pathway analysis. Among the top 10 items, the Wnt/β-catenin pathway was simultaneously activated in the three groups (Fig. 4e). Because the three Fbxw11 variants showed similar effects on the cell cycle, Gene Ontology (GO) and Kyoto Encyclopedia of Genes and Genomes (KEGG) analyses were performed based on the genes within the intersecting set shown in Fig. 4f, g, which show the top 25 significant categories in GO biological processes and KEGG pathways, respectively. Immune response-associated pathways, such as cytokine-mediated pathway and chemokine-mediated pathway, were enriched in both GO and KEGG analyses. Categories of regulation of cell proliferation and positive regulation of T-cell proliferation were also enriched in GO biological processes. Notably, the NF-κB signaling pathway was enriched in the KEGG analysis, consistent with our previous results.

### Fbxw11 promoted the expression of some proliferation-associated genes

**Fig. 5:**
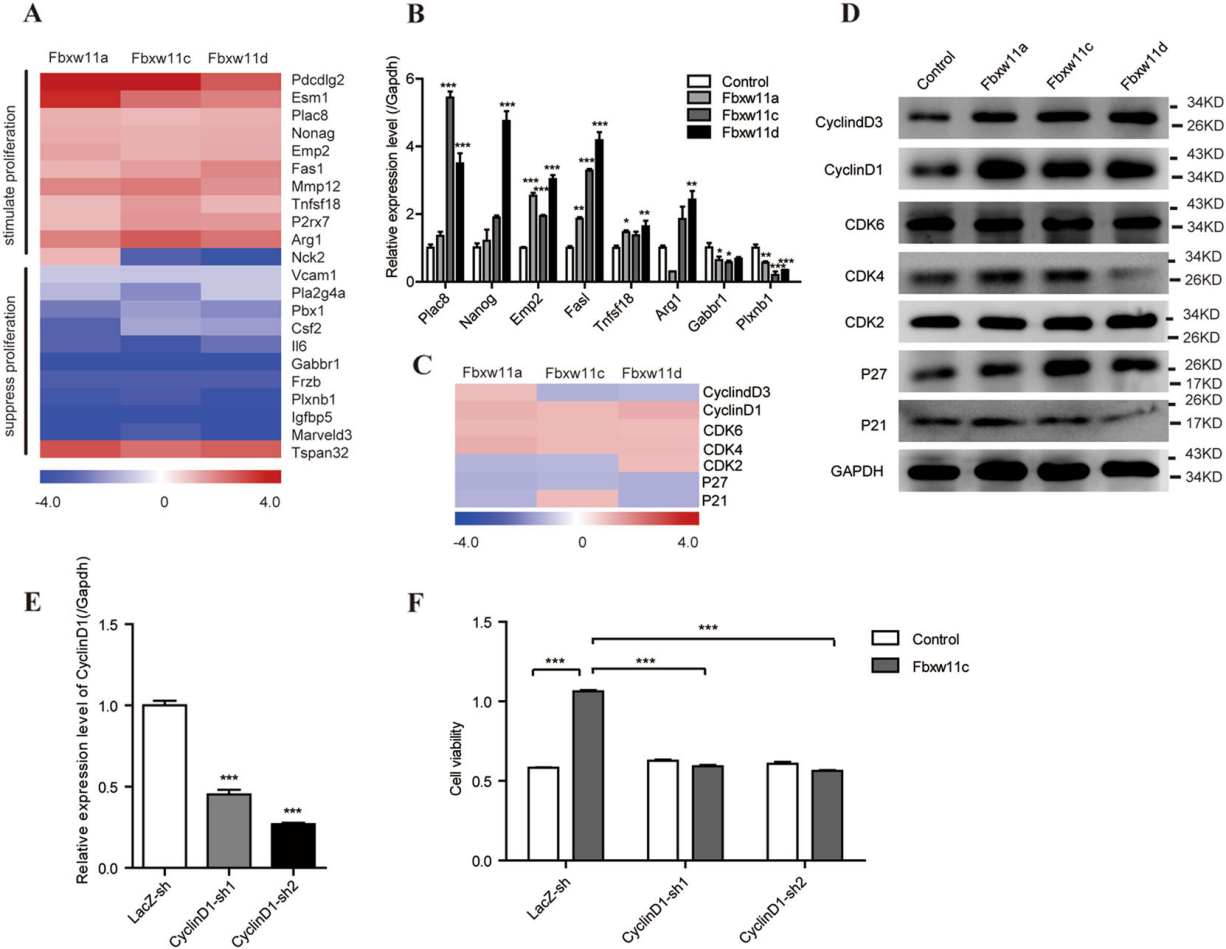
Analysis of proliferation-associated genes in L1210 cells expressing high levels of Fbxw11 variants. **a** Proliferation-associated genes, including 13 proliferation-promoting genes and 9 proliferation-suppressing genes, were selected. The data were standardized to L1210-control cells. **b** The expression of these genes was validated by real-time PCR. **c** Genes regulating the cell cycle were also selected for clustering and normalized to L1210-control cells. **d** Western blots were performed to detect the expression of eight integral proteins regulating the cell cycle. GAPDH was used as control. **e** Real-time PCR analysis showed that transfection of cells with Cyclin D1-specific shRNAs significantly reduced its mRNA levels in L1210 cells. **f** Cyclin D1 silencing decreased the proliferation of L1210 cells over-expressing Fbxw11c. Data are presented as means ± SEM (*n* = 3; 3 independent experiments). *p < 0.05, **p < 0.01, ***p < 0.001

Based on the results of the GO analysis of biological processes, the category associated with proliferation was selected for further analysis, and the selected genes are listed in Fig. 5a. Thirteen of these genes were reported to stimulate proliferation, and 9 were reported to suppress proliferation in previous studies. Plac8, Nanog, Emp2, Fasl, Tnfsf18, Arg1, Gabbr1, and Plxnb1 were selected and verified by real-time PCR. With the exception of Arg1 in L1210-Fbxw11a cells, the expression of proliferation-promoting genes was upregulated while the expression of proliferation-suppressing genes was downregulated in L1210-Fbxw11a/c/d cells (Fig. 5b). These results were consistent with the gene array data. Cell cycle checkpoint genes were also analyzed to further examine the effects of Fbxw11 on the cell cycle. Most of these genes displayed low fold changes in the gene array data (Fig. 5c). However, levels of the Cyclin D1 protein, which is regulated by multiple transcription factors, including NF-κB and β-catenin, were significantly increased in L1210 cells expressing high levels of Fbxw11 transcript variants, according to the Western blot analysis (Fig. 5d). We designed a cyclin D1 shRNA to reverse of the effect of Fbxw11 on proliferation and validate the role of Cyclin D1 on the Fbxw11-induced increase in cell proliferation (Fig. 5e). As expected, knockdown of cyclin D1 decreased the proliferation of L1210 cells (Fig. 5f).

### Fbxw11 might promote cell proliferation by activating both the NF-κB and β-catenin/TCF signaling pathways

**Fig. 6:**
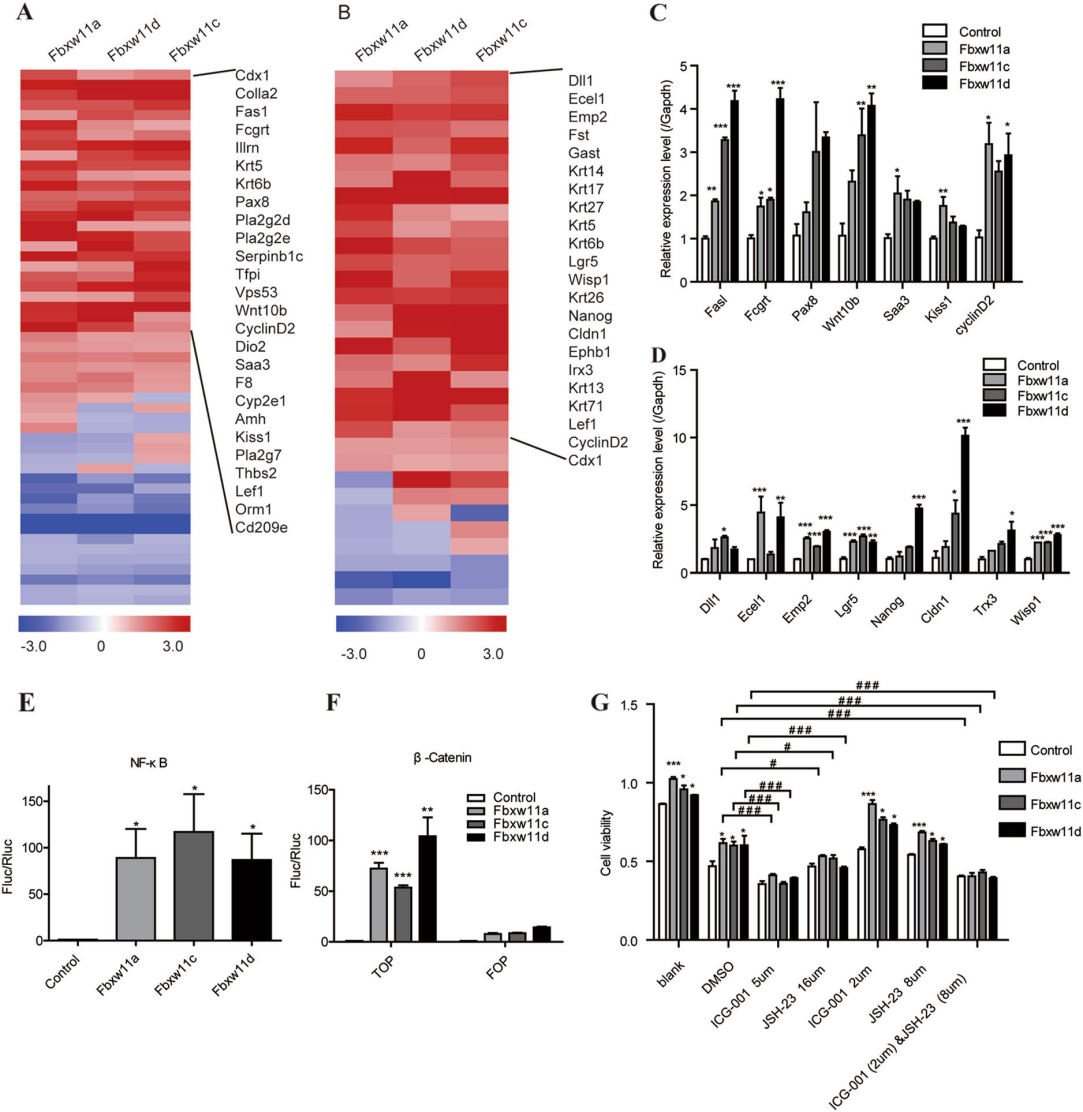
Fbxw11 activated both the NF-κB and β-catenin/TCF signaling pathways. **a**, **b** Genes downstream of the NF-κB and β-catenin/TCF signaling pathways were selected for clustering and normalized to L1210-control cells. **c**, **d** The expression of representative genes regulated by the NF-κB and β-catenin/TCF signaling pathways were verified by real-time PCR. **e** The dual luciferase method was used to analyze the activation of the NF-κB pathway in HEK293T cells expressing high levels of the Fbxw11 transcript variants. The control was set to 1. **f** The dual luciferase method was used to analyze the activation of the β-catenin/TCF signaling pathway in HEK293T cells expressing high levels of Fbxw11 transcript variants, and a mutant TCF transcription factor (FOP) was used as a functional control. The controls were set to 1. **g** The effects of NF-κB and β-catenin/TCF signaling pathway inhibitors on the proliferation of leukemia cells. Data are presented as means ± SEM (*n* = 3; 3 independent experiments). *Compared to control cells, *p < 0.05, **p < 0.01, ***p < 0.001;^ #^Compared to DMSO, ^#^*p* < 0.05, ^###^*p* < 0.001

As Cyclin D1 is downstream of both the NF-κB and β-catenin/TCF signaling pathways, we must determine which pathway is activated during the Fbxw11-mediated increase in proliferation. Hence, DEGs downstream of either NF-κB or β-catenin/TCF signaling pathway are plotted in Fig. 6a, b, respectively. The genes upregulated by Fbxw11 were summarized. Among these genes, upregulation of seven NF-κB-regulated genes in L1210-Fbxw11a/c/d cells was verified by real-time PCR, although slight differences in magnitude were observed among the three groups (Fig. 6c). Eight β-catenin/TCF-regulated genes were also verified by real-time PCR. The expression of Emp2, Lgr5, and Wisp1 was upregulated in L1210-Fbxw11a/c/d cells. Dll1, Ecel1, Nanog, Cldn1, and Trx3 were upregulated to various extents in L1210 cells expressing high levels of Fbxw11 transcript variants (Fig. 6d). A dual luciferase reporter system was also used. Activation of both the NF-κB and β-catenin/TCF signaling pathways was detected in 293T cells expressing high levels of Fbxw11 transcript variants (Fig. 6e, f). ICG-001 and JSH-23, inhibitors of the NF-κB and β-catenin/TCF pathways, were used to further confirm that both the NF-κB and β-catenin/TCF signaling pathways mediate the pro-proliferation effects on L1210-Fbxw11a/c/d cells. The administration of high doses of both inhibitors (5 μM for ICG-001 and 16 μM for JSH-23) completely diminished the pro-proliferation effects of Fbxw11 on L1210 cells. Furthermore, the administration of a combination of low doses of inhibitors (2 μM for ICG-001 and 8 μM for JSH-23) completely diminished the pro-proliferation effects of Fbxw11, although treatment with a low dose of either inhibitor had little effect (Fig. 6g). Based on the results of the apoptosis analysis, all dosages used in the experiments displayed little toxic effects (Figure S4). The above data suggested that Fbxw11 promoted the proliferation of L1210 cells by concomitantly activating the NF-κB and β-catenin/TCF signaling pathways.

## Discussion

The genesis and progression of leukemia are multifactorial processes that are regulated by a combination of microenvironmental and internal factors. The UPS is an important protein control system that participates in many physiological processes, such as cell proliferation, differentiation, and signal transduction. Dysfunction of ubiquitination or abnormal expression of UPS components is closely related to the occurrence of a variety of tumors and leukemia^16,20^. In our previous study, we observed upregulation of Fbxw11 in HSPCs in a T-ALL microenvironment^36^. However, little is known about the effects of Fbxw11 on leukemia cells. In the present study, Fbxw11 was aberrantly upregulated in patients with certain subtypes of ALL. Furthermore, high levels of Fbxw11 expression stimulated L1210 cell proliferation by regulating the cell cycle process. Moreover, both the NF-κB and β-catenin/TCF signaling pathways were activated in this process. Our results not only elucidate the significance of the Fbxw11-associated UPS in the progression of ALL but also provide new insights for leukemia research and clinical therapy.

The correlation between high levels of Fbxw11 expression and tumors has been reported in skin tumors and colorectal cancer, among others^33,37^. In our study, Fbxw11 was expressed at higher levels in patients with newly diagnosed and relapsed ALL than in healthy control donors. The E3 ligases Fbxl2, Fbxl10, and SKP2 participate in the proliferation of leukemia cells^20–22^. According to previous studies, β-TrCP (including β-TrCP1 and Fbxw11) is involved in regulating cell division^38^. Here we detected the growth-promoting effects of Fbxw11 variants on L1210 cells both in vitro and in vivo. Based on the results of the apoptosis and cell cycle analyses, the effects were due to stimulation of the cell cycle rather than the induction of apoptosis. The bioinformatics analysis of microarray data showed that categories involved in the regulation of cell proliferation and positive regulation of T-cell proliferation were enriched in the GO biological process analysis. Furthermore, the upregulation of proliferation-associated genes was also validated. These results further confirmed that high levels of Fbxw11 expression promoted the proliferation of ALL cells, and three Fbxw11 variants exerted similar effects.

The mechanism by which Fbxw11 stimulated the proliferation of ALL cells was of interest. Enhanced constitutive activation of the NF-κB pathway has been observed in several human tumor cells. Activation of the NF-κB pathway upregulates cyclins, such as cyclin D1^39^. Moreover, a series of inhibitors of the NF-κB pathway are degraded by F-box proteins, including Fbxw11^26,28,40,41^. In our study, the category of the NF-κB signaling pathway had a high enrichment score in the KEGG analysis. Furthermore, a reporter gene assay provided direct evidence that high levels of Fbxw11 expression enhanced the transcriptional activity of the NF-κB signaling pathway. Moreover, upregulation of cyclin D1 was detected in L1210 cells expressing high levels of Fbxw11 variants. These results suggested that the NF-κB-cyclinD pathway was activated by Fbxw11 to stimulate the proliferation of ALL cells.

The β-catenin/TCF signaling pathway is implicated in many types of human tumors^23^. Accumulation and nuclear translocation of β-catenin leads to the activation of TCF transcription factors, followed by the induction of transcription of a number of proliferation-associated genes, including cyclin D1^42^. Fbxw11 recognizes β-catenin and is involved in its degradation^26,43^. In our study, the Wnt/β-catenin pathway was simultaneously activated in the three groups expressing high levels of Fbxw11 variants in the IPA analysis (Fig. 4f). The reporter gene assay further confirmed that Fbxw11 overexpression promoted the transcriptional activity of the β-catenin/TCF signaling pathway. The mechanism is not clear. However, a similar phenomenon has also been observed for other F-box proteins. For example, EDD3, FANCL, and RAD6B interact with β-catenin, leading to their ubiquitination. However, high levels of those F-box proteins increase the level of the β-catenin protein and the activation of the β-catenin pathway^44–46^. These results suggest that β-catenin/TCF signaling pathway is also activated by Fbxw11 to stimulate the proliferation of leukemia cells.

Studies on the mechanism of Fbxw11 in malignant cells in the literature have mainly focused on either the NF-κB or β-catenin signaling pathway^23,47^. However, both of these pathways converge to regulate the activity of the cyclin D1 gene promoter^39,47,48^. Intriguingly, both the NF-κB and β-catenin/TCF signaling pathways were activated in L1210 cells expressing high levels of Fbxw11 in the present study. Inhibitors of the two signaling pathways were used to test their effects on cell proliferation and further confirm the concomitant involvement of the two pathways. Treatment with a combination of low doses of the inhibitors completely diminished the pro-proliferation effects of Fbxw11, although treatment with a low dose of either inhibitor alone had little effect. These results suggest that increased activities of the SCF^Fbxw11^ E3 ubiquitin ligase in ALL cells promotes cell proliferation by accelerating cell cycle events through concomitant activation of the NF-κB and β-catenin/TCF signaling pathways.

Taken together, Fbxw11 expression is upregulated in patients with ALL. High levels of Fbxw11 expression stimulate the proliferation of L1210 lymphocytic leukemia cells in vitro and promote tumor formation in vivo by regulating the cell cycle. Most importantly, activation of both the NF-κB and β-catenin/TCF signaling pathways is involved in this process. This work reveals the role of Fbxw11 in the proliferation of lymphocytic leukemia cells and implies that Fbxw11 may serve as a potential molecular target for the treatment of lymphocytic leukemia.

## Materials and methods

### Leukemia samples

BM samples were obtained from pediatric patients with AL who were aged <18 years at the initial diagnosis from the Blood Diseases Hospital, Chinese Academy of Medical Sciences & Peking Union Medical College. Patients included 60 individuals with newly diagnosed ALL, 59 with newly diagnosed acute myelocytic leukemia, 12 with relapsed ALL, and 15 with ALL in CR. Among the 15 patients with ALL in CR, only 7 cases had samples at newly diagnosed stage and they were also included in the 60 newly diagnosed ALL group. These seven cases were chosen for follow-up study to compare Fbxw11 expression in patients before and after standard chemotherapy achieving CR. Bone marrow mononuclear cells were obtained by Ficoll-Hypaque density gradient centrifugation. Approval was obtained from the Institutional Research Board at Blood Disease Hospital prior to the initiation of this study.

### Reagents and vectors

PRMI-1640, fetal bovine serum (FBS), and M-MLV reverse transcriptase were purchased from Thermo Fisher Scientific (Carlsbad, CA). The SYBR Premix Ex Taq Kit was obtained from TaKaRa Biotech (Dalian, China). The monoclonal antibody against mouse Fbxw11 was purchased from Sigma-Aldrich (St. Louis, MO). The enhanced chemiluminescence detection kit was purchased from Millipore (Bedford, MA). The lentivirus-based vector pLV-EF1α-MCS-IRES-Bsd (Cat# cDNA-pLV03) and pLV-H1-EF1α-red (Cat# SORT-B11) were obtained from Biosettia Inc. (San Diego, CA).

### Cell lines

The L1210 cell line was purchased from ATCC and stored at the cell bank of the State Key Laboratory of Experimental Hematology (SKLEH). L1210 cells were infected with lentiviruses carrying Fbxw11 variants. After sorting by flow cytometry, the GFP^+^ stably transfected cell lines were named L1210-control, L1210-Fbxw11a, L1210-Fbxw11c, and L1210-Fbxw11d, respectively. All cells were maintained in RPMI 1640 medium supplemented with 10% FBS, glutamine (2 mM), penicillin (100 U/ml), and streptomycin (100 μg/ml), at 37 °C in 5% CO_2_ atmosphere.

### cDNA synthesis and real-time RT-PCR

The cDNA templates were prepared using a previously described method^49^. Briefly, total RNA was extracted using Trizol Reagent (Invitrogen, Carlsbad, CA, USA) and cDNAs were then synthesized using M-MLV reverse transcriptase (Invitrogen), according to the manufacturer’s instructions. Real-time PCR was performed using an ABI 7500 Sequence Detector System (Applied Biosystems, Foster City, CA). The expression level of target genes was obtained from at least three independent experiments by calculating the RQ value using the ^ΔΔ^Ct method [^ΔΔ^Ct = (Ct_TARGET_−Ct_GAPDH_) sample−(Ct_TARGET_−Ct_GAPDH_) calibrator]. The sequences of all primers are listed in Table 1 in the supplementary materials. For each gene, the RQ value of L1210-control was designated 1.00.

### Western blot

Total protein was extracted from cells using lysis buffer (Cell Signaling Technology, Danvers, MA) supplemented with protease inhibitors and phenylmethanesulfonylfluoride. Separation on sodium dodecyl sulfate-polyacrylamide gel electrophoresis gels, transfer to polyvinylidene difluoride (PVDF) membrane and film development were performed using a standard protocol^50^. PVDF membranes were incubated with diluted primary antibodies (1:1000 for Fbxw11; QC13573, Sigma, St. Louis, MO) and 1:5000 for glyceraldehyde 3-phosphate dehydrogenase (GAPDH; 97166, Cell Signaling, Danvers, MA) in TBST buffer containing 5% milk at 4 ℃ overnight. Western blots were repeated at least three times.

### Cell cycle analysis

PI staining was used for the cell cycle analysis. Briefly, cells were harvested, suspended in phosphate-buffered saline (PBS) and fixed with 70% ethanol on ice for 1 h. Then cells were washed with PBS and RNA was digested with RNase. Haploid and diploid DNA were labeled with PI overnight. Ki-67/PI double staining was also employed to detect cells in G0 phase. Briefly, suspended cells were treated with Cytofix/Cytoperm buffer (BD Pharmingen) and stained with Ki-67-PE (Biolegend, San Diego, CA) and Hoechst33342 (Sigma). An LSRII flow cytometer was used for the cell cycle analysis according to standard protocols. Experiments were repeated at least three times.

### Mouse model

Six-to-8-week-old female DBA2 mice were purchased from Beijing Vital River Laboratory Animal Technology Co., Ltd. (Beijing, China) and maintained in the animal center of SKLEH. All experiments were approved by the Institutional Animal Care and Use Committees of SKLEH. The dorsal surface of each mouse was subcutaneously. injected with 1 × 10^6^ cells in a volume of 200 μL. Tumor volumes were monitored weekly. Mice were sacrificed 4 weeks after cells were injected. Tumor volumes were calculated using the formula length × width^2^/2.

### Immunofluorescence staining for the BrdU incorporation assay

Mice were intraperitoneally injected with 50 mg/kg BrdU 16 h prior to sacrifice. Tumors were isolated, fixed with 4% paraformaldehyde for 24 h, and then 5-µm-thick paraffin-embedded tissue sections were generated. Tissue sections were stained with an anti-BrdU antibody (Cell Signaling Technology, Danvers, MA) followed by DyLight^TM^ 649-conjugated goat anti mouse IgG (Biolegend, San Diego, CA) to determine the extent of BrdU incorporation in tumors. DAPI (4,6-diamidino-2-phenylindole) was used to counterstain the nuclei. Sections were scanned using a confocal laser scanning microscope (UltraView Vox, PerkinElmer, MA).

### Microarray and data analysis

First, mRNAs were extracted from L1210-control, L1210-Fbxw11a, L1210-Fbxw11c, and L1210-Fbxw11d cells, respectively. An Agilent SurePrint G3 Mouse Gene Expression V 2.0 microarray was analyzed by Shanghai OE Biotech. Co., Ltd. using standard protocols. The microarray data are available in the National Center for Biotechnology Information Gene Expression Omnibus database under accession number GSE101725. DEGs in L1210-Fbxw11a, L1210-Fbxw11c, and L1210-Fbxw11d cells were normalized to L1210-control cells and filtered with a fold change ≥2.0 to obtain the three groups of DEGs. The intersections of these DEGs were analyzed by performing GO and KEGG pathway analyses using the FunNet online database. The significance of the gene enrichment in the considered GO and KEGG categories was calculated using a unilateral Fisher exact test (*p*-value) and a false discovery rate <0.01. The sum of these three groups of DEGs was also analyzed by a comparison analysis using the IPA software, and the common canonical pathway was obtained based on the activation *z*-score. The expression patterns among the different groups were analyzed using the MeV 4.9.0 software.

### Knockdown Cyclin D1 by shRNA

shRNA sequences for silencing mouse Cyclin D1 were designed using RNAi designer from ThermoFisher website (http://rnaidesigner.thermofisher.com/rnaiexpress/index.jsp). The two shRNA sequences were synthesized as Cyclin D1-sh1 (AAAAGCTGCAAATGGAACTGCT TCTTTGGATCCAAAGAAGCAGTTCCATTTGCAGC) and Cyclin D1-sh2 (AAAAGGAA CAGATTGAAGCCCTTCTTTGGATCCAAAGAAGGGCTTCAATCTGTTCC). An shRNA targeting LacZ (AAAAGCAGTTATCTGGAAGATCAGGTTGGATCCAACCTGATCTTCCAGATAACTGC) was used as control for knockdown analysis. The single-stranded DNA oligos were annealed to form a double-strand oligos and ligated to the linearized pLV-H1-EF1α-red vector to construct shRNA vectors. L1210-control and L1210-Fbxw11c cells were infected with lentiviruses carrying different shRNA vectors, respectively. After sorting by flow cytometry, the RFP^+^ cells were used for further analysis. The efficacy of knockdown by shRNA was verified by real-time RT-PCR.

### Statistical analysis

All quantitative data are presented as the means ± SEM and were analyzed using one-way analysis of variance (ANOVA) and followed by Dunnett’s post hoc multiple comparison test (to compare all columns with the control column). Two-way ANOVA followed by Bonferroni’s post hoc test was also used to analyze multiple factors. The significance of differences in Fbxw11 expression between ALL specimens and ALL-CR specimens was tested using a paired *t*-test. Data were analyzed using the GraphPad Prism software. Two-sided *p*-values <0.05 were considered statistically significant.

## Electronic supplementary material

Figure S1(TIF 6211 kb)

Figure S2(TIF 586 kb)

Figure S3(TIF 6552 kb)

Figure S4(TIF 13179 kb)

Supplemental figure legend(DOCX 12 kb)

Table 1(DOCX 16 kb)
