# Supplementary figures and images for: Fbxw11 promotes the proliferation of lymphocytic leukemia cells through the concomitant activation of NF-κB and β-catenin/TCF signaling pathways
Source: Cell Death Dis. 2018 Mar 19;9(4):427. doi: 10.1038/s41419-018-0440-1 (PMC5859049; doi:10.1038/s41419-018-0440-1)

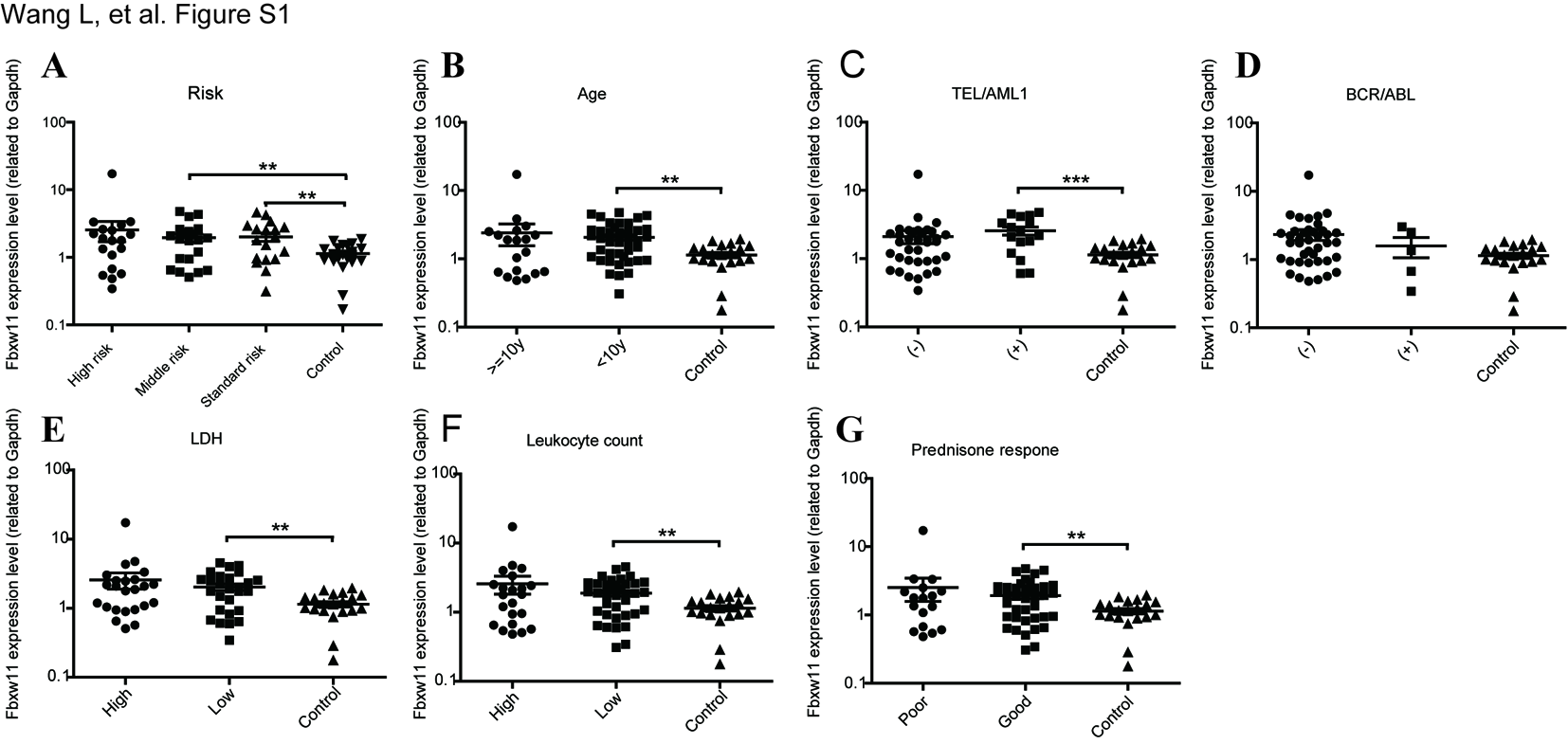

Supplement: Supplementary file 1 — Figure S1(TIF 6211 kb) [file 41419_2018_440_MOESM1_ESM.tif]

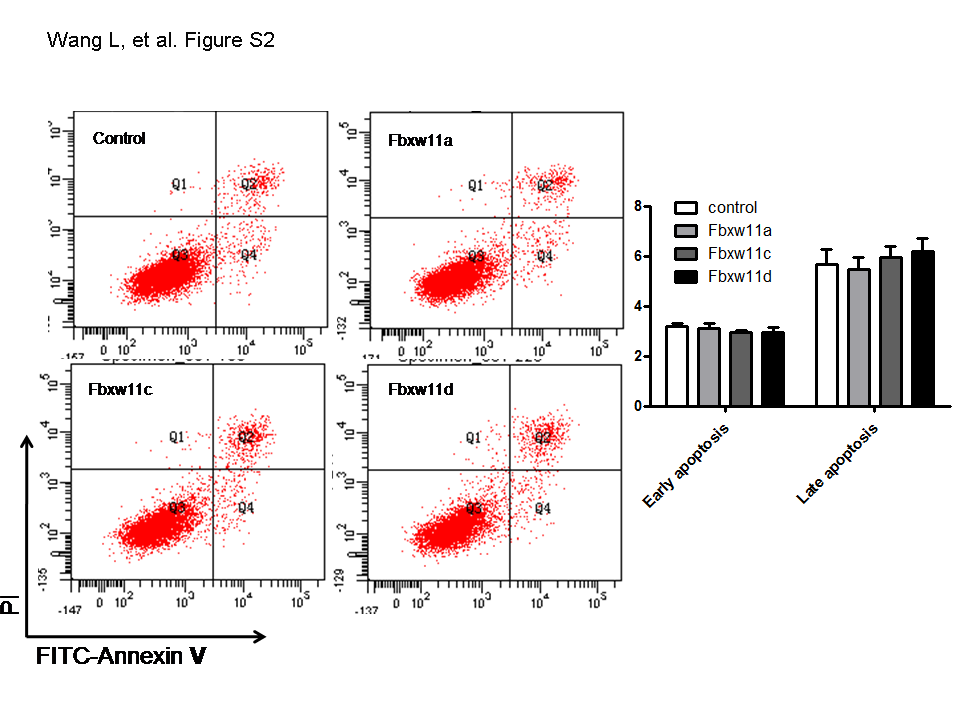

Supplement: Supplementary file 2 — Figure S2(TIF 586 kb) [file 41419_2018_440_MOESM2_ESM.tif]

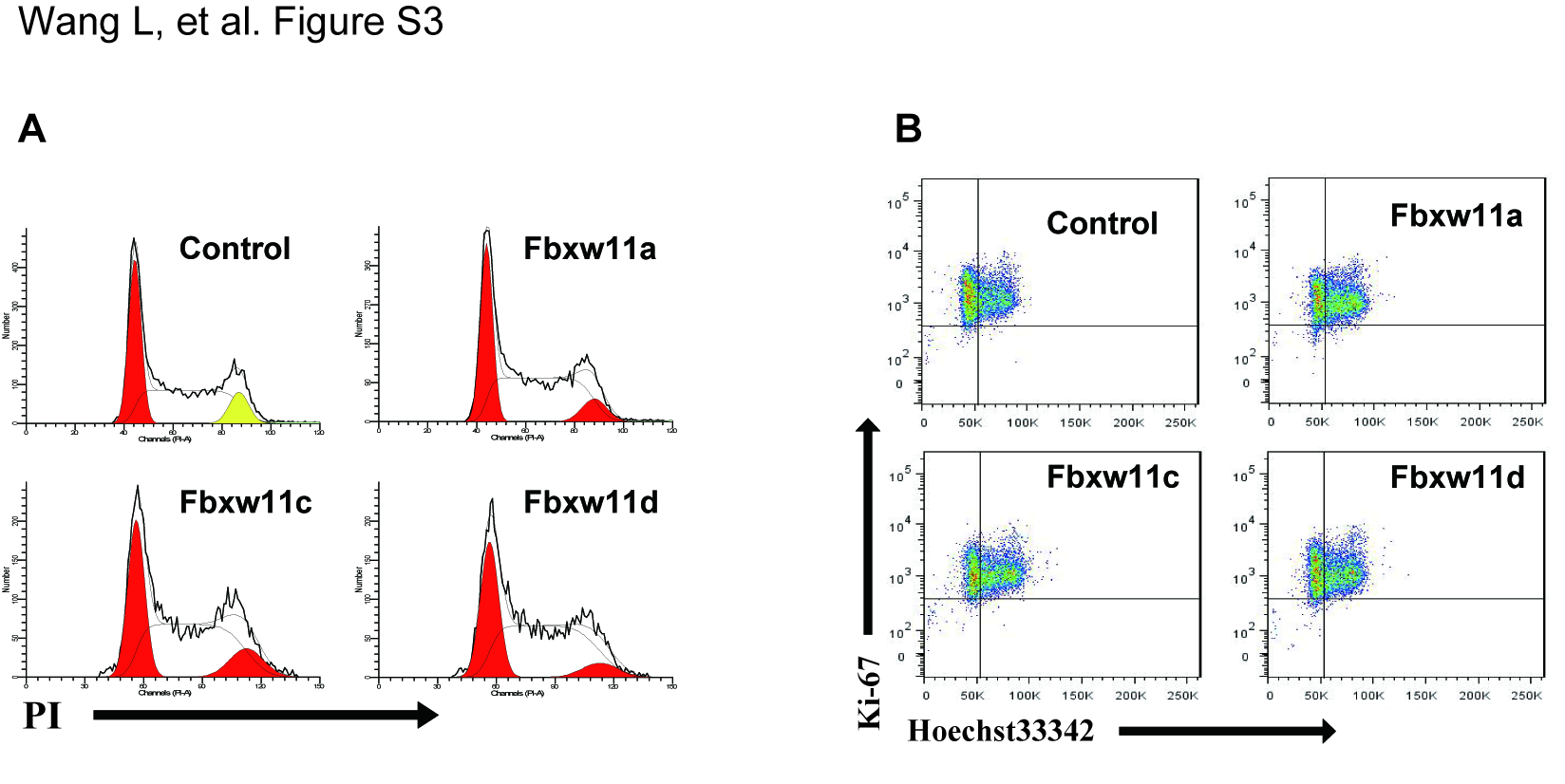

Supplement: Supplementary file 3 — Figure S3(TIF 6552 kb) [file 41419_2018_440_MOESM3_ESM.tif]

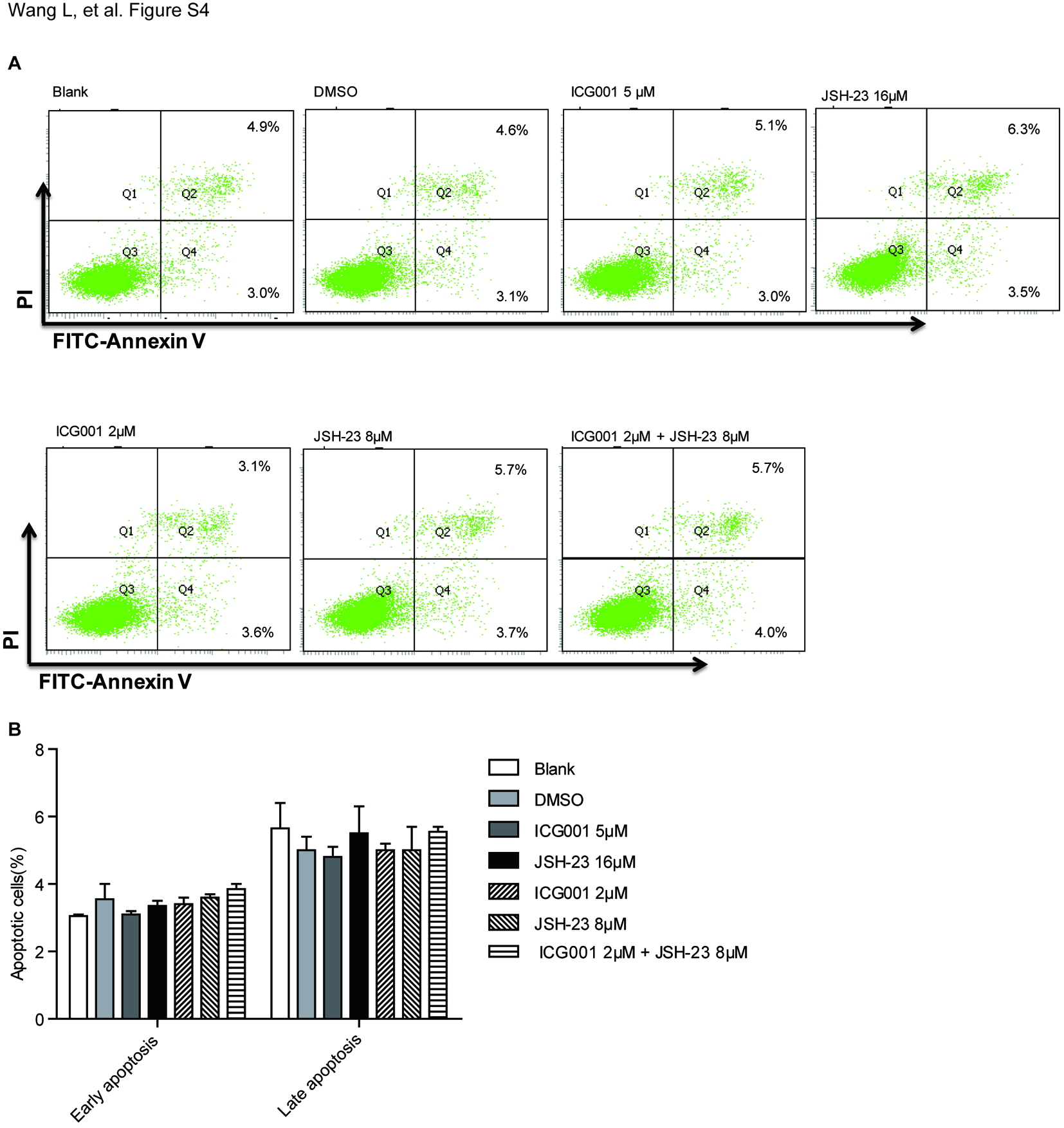

Supplement: Supplementary file 4 — Figure S4(TIF 13179 kb) [file 41419_2018_440_MOESM4_ESM.tif]
