# Supplementary material for: Fbxw11 promotes the proliferation of lymphocytic leukemia cells through the concomitant activation of NF-κB and β-catenin/TCF signaling pathways
Source: Cell Death Dis. 2018 Mar 19;9(4):427. doi: 10.1038/s41419-018-0440-1 (PMC5859049; doi:10.1038/s41419-018-0440-1)
Supplement: Supplementary file 5 — Supplemental figure legend(DOCX 12 kb) [file 41419_2018_440_MOESM5_ESM.docx]

**Figure S1.** Analysis of Fbxw11 expression and clinical features in newly diagnosed pediatric ALL. Patients were grouped according to different clinical features. Risk group: high-risk, middle-risk and standard-risk; Age: ≥10 years and 1–10 years; TEL/AML1: (-) and (+)； BCR/ABL: (-) and (+)； LDH： High and low (groups with LDH level P≥500 and <500 U/L). Leukocyte counts： High and Low (groups with leukocyte counts ≥50 and <50×109/L when newly diagnosed)； Prednisone response: Poor and good (groups with poor and good prednisone response after hormonal therapy on 8 days.

**Figure S2.** Effect of Fbxw11 on cell apoptosis of L1210 cells. L1210 cells with overexpression of Fbxw11 transcript variants were harvested and stained with PI and Annexin V to be subjected to flow cytometry analysis.

**Figure S3** (A) Representative FACS results from cell cycle analysis by PI staining. (B) Representative FACS results from cell cycle analysis by Ki67 and hoechst 33342 staining.

**Figure S4** (A) Representative FACS results of apoptosis to analyze cytotoxic effect of the high concentration of drugs. (B) Quantitative analysis of the apoptosis assay. There was no difference in apoptosis among different drug treatment.
