# Supplementary material for: Fbxw11 promotes the proliferation of lymphocytic leukemia cells through the concomitant activation of NF-κB and β-catenin/TCF signaling pathways
Source: Cell Death Dis. 2018 Mar 19;9(4):427. doi: 10.1038/s41419-018-0440-1 (PMC5859049; doi:10.1038/s41419-018-0440-1)
Supplement: Supplementary file 6 — Table 1(DOCX 16 kb) [file 41419_2018_440_MOESM6_ESM.docx]

**Table 1** Primers used for detection of human and mouse genes by real-time RT-PCR analysis

| Gene name | Forward primer | Reverse primer |
| --- | --- | --- |
| Fbxw11(homo) | GTGGGATGTGAACACGGGTGA | CGTAAAGTGATGTCGGTCGCAG |
| Fbxw11(mus) | CGGGACTTTATCACTGCTTTA | ATCACTCGCTGCCATTCTTTA |
| GAPDH(homo) | GAAGGTGAAGGTCGGAGTC | GAAGATGGTGATGGGATTTC |
| GAPDH(mus) | CACTTGAAGGGTGGAGC | GGGCTAAGCAGTTGGTG |
| Plac8(mus) | ATGGCTCAGGCACCAACAGT | GCCGTATCGGGTTCGGTAGA |
| Nanog(mus) | TCTCCTCGCCCTTCCTCTGA | GAGAGAACACAGTCCGCATCTT |
| Emp2(mus) | TTGACAATGCCTGGTGGGTA | GCTGGAGCAGAAAGATGAG |
| Fasl(mus) | CACTTCATCTTGGGCTCCTC | TGTTGTGGTCCTTCTTCTTT |
| Tnfsf18(mus) | TCATGGCTCTTGTGCATAGT | TTCCCATCAGATGTCGTATT |
| Arg1(mus) | CAACCAGCTCTGGGAATCTG | AATCGGCCTTTTCTTCCTTC |
| Fcgrt(mus) | TTCCCTCTTTCTGGGCTACA | TTCTCCCAATACCAAGACACCT |
| Pax8(mus) | GCAGCAGTGGTCCTCGAAAG | TGTTGAGTAAGGGCAGTGGG |
| Wnt10b(mus) | GGTGGGACGCCAGGTGGTAA | AGTTGCGGTTGTGGGTATCG |
| Saa3(mus) | GGCTGTTCAGAAGTTCACGG | AGCAGGTCGGAAGTGGTTGG |
| Kiss1(mus) | CTCGTAGGTCGTCGCCATGC | GAAGGAGTTCCAGTTGTAGG |
| CyclinD2(mus) | CTGCTGTGCTGCGAGGTG | AGCCAAGAAACGGTCCAGGTAA |
| Dll1(mus) | CAATGGAGGACGATGTTCAGA | ACAGGTAAGAGTTGCCGA |
| Ecel1(mus) | CTATCTGCCCAACAAGAATCAA | TCTCAGTCCACCAGTGCAACA |
| Emp2(mus) | TTGACAATGCCTGGTGGGTA | GCTGGAGCAGAAAGATGAG |
| Lgr5(mus) | GGGAACCGAGCCTTACAG | CCCGCTCATCTTGAACTTGAAATA |
| Cldn1(mus) | CTGGGTTTCATCCTGGCTTCT | CTGTATCTGCCCGGTGCTT |
| Irx3(mus) | GACACGGGGGGCGAGGGGCT | CGAAGTCGCCGTTCCTGTGC |
| Wisp1(mus) | GTGATGATGACGCAAGGAGAC | CGGGCATTGACGTTAGAGA |
| Gabbr1(mus) | TTAGGCTTTAGTCTGGGCTATG | AAGTCTCAATGGTTCGGTGC |
| Plxnb1 | GGGCACCTTATCCTTTCTGA | CCTCATCTACATCCTCCAGCA |
